# Clinical Cases of Coccidioidomycosis in the Americas in the Period 1950–2021: Epidemiology Data, Diagnosis, and Treatment

**DOI:** 10.3390/life13112109

**Published:** 2023-10-24

**Authors:** María del Rocío Reyes-Montes, Adriana Anel León-García, María Guadalupe Frías-De-León, Gustavo Acosta-Altamirano, Erika Paola Sánchez-Saavedra, Isai Victoriano-Pastelín, Beatriz Meraz-Ríos, Esperanza Duarte-Escalante

**Affiliations:** 1 Departamento de Microbiología y Parasitología, Facultad de Medicina, Universidad Nacional Autónoma de México, Ciudad Universitaria, Coyoacán, Ciudad de Mexico 04510, Mexico; remoa@unam.mx (M.d.R.R.-M.); panny.garcia.pg@gmail.com (A.A.L.-G.); erikapssaavedra@gmail.com (E.P.S.-S.); cafei_nternet@hotmail.com (I.V.-P.); bmerazr@hotmail.com (B.M.-R.); 2 Hospital Regional de Alta Especialidad de Ixtapaluca, Unidad de Investigación Biomédica, Pueblo de Zoquiapan, Ixtapaluca 56530, Mexico; magpefrias@gmail.com (M.G.F.-D.-L.); mq9903@live.com.mx (G.A.-A.)

**Keywords:** coccidioidomycosis, treatment, diagnosis, clinical cases

## Abstract

Coccidioidomycosis, caused by *Coccidioides immitis* and *C. posadasii*, causes significant morbidity and mortality, both in immunocompetent and immunocompromised people, mainly in endemic areas. The present work analyzed its epidemiology, diagnostic methods, and treatment by reviewing clinical cases published from 1950 to 2021. Fifty-nine articles were included, corresponding to 275 clinical cases. The results showed a higher incidence of coccidioidomycosis in the male gender than the female gender. The most affected age group was 31–40 years, and the most reported clinical presentation was disseminated with greater involvement in cutaneous and subcutaneous tissue, followed by the CNS, bone system, and peritoneum. The species most frequently reported was *C. immitis*. The most used treatment was azoles, followed by their combination with amphotericin B, monotherapy with amphotericin B, and alternative medicine. This work shows that epidemiological data outside the USA are still scarce. Serological tests are the preferred diagnostic method in daily medical practice, and cultures remain the gold standard. The treatment for coccidioidomycosis is ketoconazole and amphotericin B, individually or in combination.

## 1. Introduction

Coccidioidomycosis is a disease caused by the fungi *Coccidioides immitis* and *C. posadasii* and is considered endemic to the American continent. From an epidemiology standpoint, the most important areas include the southwestern United States, northern Mexico, Central America, and some parts of South America [1,2].

This mycosis causes significant morbidity and mortality, both in immunocompetent and immunocompromised people worldwide, mainly in endemic areas. Nevertheless, each region has specific epidemiological data. For instance, it has been shown that the incidence of coccidioidomycosis in the USA increased during the last two decades, both in endemic and non-endemic regions within the country. The Centers for Disease Control and Prevention (CDCs) reported a 58% increase in the incidence of coccidioidomycosis in Arizona from October 2017 to March 2018 compared to previous years, with California being the state with the highest reporting in 2017 [2]. However, it is worth noting that this disease does not require mandatory notification on the rest of the American continent, except Argentina. Therefore, data on the incidence of coccidioidomycosis are not accurate, and the information is limited to reports of disseminated or unusual clinical cases [1,2,3,4]. In Mexico, *Coccidioides* infection is considered to be as prevalent as in the endemic regions of the USA, with an average of 1500 cases reported per year, according to data reported from 1988 to 1994, where the mean incidence of coccidioidomycosis was 0.8 to 10.5 [1]. Still, this mycosis ceased to require mandatory notification as of 1994. Meanwhile, in Argentina, epidemiological studies have revealed the highest infection rates in the province of Catamarca (40%). Other areas with high infection rates are northwestern Córdoba (34%), the western part of Santiago del Estero (19.8%), and La Rioja (19.13%), which are neighboring provinces of Catamarca [5]. On the other hand, reports are scarce in Central American countries such as Guatemala and Honduras, even though the fungus has been noted. Likewise, in other South American countries, the incidence of this mycosis is unknown [1].

Typically, the infection begins when a susceptible host inhales the arthroconidia, considered the infective phase, present in the soil or air. Once in the host, they become spherules (parasitic phase) containing abundant endospores, and each of these can produce another spherule, thus remaining in the host [6]. 

**Clinical forms.** Coccidioidomycosis is divided into three categories: primary pulmonary form, progressive pulmonary form, and disseminated form. In primary pulmonary coccidioidomycosis, it is estimated that 60% of patients have asymptomatic infections that can only be detected by serological or skin tests when symptoms appear. Additionally, coccidioidomycosis infection’s primary respiratory signs and symptoms may be indistinguishable from those of common bacterial pneumonia. Likewise, in endemic regions, coccidioidal pneumonia can represent up to 29% of community-acquired pneumonia [7,8]. On the other hand, some patients may present with erythema nodosum or erythema multiforme, which are considered markers of favorable prognosis and occur more frequently in women [9]. Spontaneous regression of primary respiratory infections is also reported, even without antifungal treatment [10,11]. Progressive pulmonary coccidioidomycosis is generally chronic and develops after the first infection, the symptoms of which do not resolve after two months [12]. Disseminated coccidiomycosis is a rare clinical entity that develops in less than 5% of immunocompetent patients; however, dissemination is common in immunocompromised hosts, pregnant patients, and patients of African and Filipino ancestry. Furthermore, immunosuppression can be caused by different factors (administration of immunosuppressive drugs, organ transplants, cancer, chemotherapy, glucocorticoid administration, and AIDS), which can increase the possibility of acquiring severe forms of the infection. Another organ of dissemination most frequently related to it is the skin; in addition to the involvement of the central nervous system, this is the most serious form and occurs in 90% of these cases [13]. Meningitis is the most common clinical presentation; however, the musculoskeletal system, lymph nodes, and pericardium have also been reported to be dissemination sites [14].

It is crucial to mention that people who become infected and are asymptomatic show mild respiratory symptoms that can be confused with a cold; therefore, patients do not seek medical care. In contrast, cases that progress with moderate or severe lung disease can be mistaken for other pathologies of bacterial etiology, such as pneumonia and tuberculosis, making the diagnosis difficult [15]. Despite the diversity of tools that can be employed for diagnosing this disease, the methods are not available for daily use in clinical laboratories, which poses a challenge. The most commonly used laboratory tools for diagnosing and treating this mycosis are mentioned below.

**Diagnosis coccidioidomycosis.** The clinical laboratory plays a critical role in diagnosing coccidioidomycosis. It is relevant to mention that cultures are considered the gold standard method for diagnosing coccidioidomycosis; however, they should only be performed in Biosafety Level 3 laboratories (BSL-3s).

Cytology is another diagnostic option performed from sputum samples, bronchoalveolar lavage, or biopsy in cases of extrapulmonary involvement, in which mainly pathognomonic parasitic forms can be observed (spherules) [16,17,18,19].

Immunodiffusion (ID), or Ouchterlony double immunodiffusion (DID), is performed based on the antibodies’ and antigens’ ability to migrate in a semisolid matrix. It has been shown that this test can be very sensitive and specific for detecting distinct antibodies against *Coccidioides*, with a specificity greater than 95% [20,21].

Another method is the complement fixation test (CF), which provides a semiquantitative evaluation of coccidioidal IgG antibodies and can be performed with serum samples, cerebrospinal fluid (CSF), and other body fluids such as synovial and pleural fluid [22]. Complement fixation is a classic method to demonstrate the presence of antibodies in the patient’s serum.

Another test is the enzyme immunoassay (EIA), which is widely available and provides timely results. Therefore, it is the most commonly used test for the initial detection of coccidioidomycosis. EIAs can be qualitative or semiquantitative. On the other hand, although detecting IgM and IgG antibodies using EIA provides better sensitivity than other available tests for identifying early disease (ID and CF), it is less specific. It is also hampered by the possibility of false-positive results [23]. Therefore, positive EIA results should be confirmed with another test, such as ID or CF, although these are usually negative early in the disease. Thus, follow-up testing every one to two weeks is justified in suspected cases [24].

The skin test with *Coccidioides* antigens was used from the beginning of the history of this disease for clinical and epidemiological purposes. For this test, the spherusol antigen is used, which is approved by the Food and Drug Administration (FDA), USA [25]. A positive test (induration >5 mm at 48 h) indicates a current or past infection caused by *Coccidioides*.

Furthermore, a rapid lateral-flow assay (LFA) has been developed recently to detect coccidioidal antibodies that provide results in one hour (Sōna; IMMY, Norman, OK, USA). This assay is advantageous as it can be conducted with minimal training and laboratory equipment. However, a recent study in patients with early coccidioidomycosis showed markedly lower sensitivity for the LFA than the EIA test [26].

Numerous PCR-based methods have been developed and applied recently for detecting *Coccidioides* species from clinical samples and cultures (Table 1). However, only one has received FDA approval (GeneSTAT, St. George, UT, USA), granted only for bronchoalveolar and bronchial lavage samples [27].

Despite numerous laboratory tests being currently available, they clearly present limitations for basic investigation. In addition, affordability is another restriction, primarily in developing countries, where some of these options are not accessible, coupled with the lack of solid clinical data to support their use.

**Treatment.** On another subject, there is no single therapeutic scheme for coccidioidomycosis. It is recommended that treatment be individualized, especially for patients at risk of complications from severe forms of the disease. The literature shows that fluconazole and itraconazole are the most frequently used antifungals for the various manifestations of coccidioidomycosis. However, other antifungals may be an option for treatment. Among the triazoles are voriconazole, posaconazole, and isavuconazole, while amphotericin B, belonging to the group of polyenes, is also a therapeutic option [38]. It should be noted that amphotericin is currently available in various formulations: amphotericin deoxycholate B, liposomal amphotericin B, amphotericin B colloidal dispersion, and amphotericin B lipid complex, all of which are only available intravenously [39].

Thus, the present work analyzes the epidemiology of coccidioidomycosis, the diagnostic methods, and the treatment of this mycosis based on a review of coccidioidomycosis clinical cases published from 1950 to 2021.

## 2. Materials and Methods

**Figure 1:**
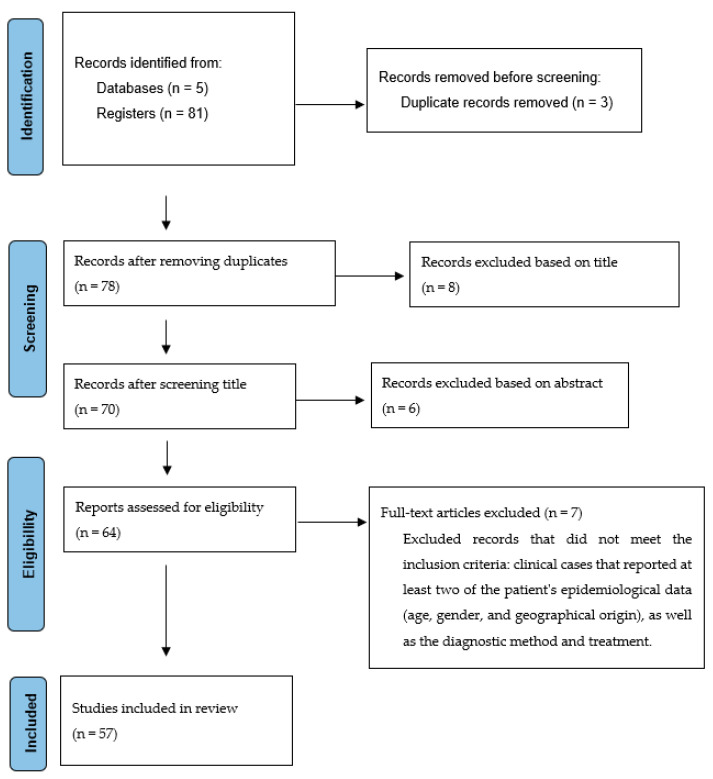
Flow diagram showing the selection of studies.

A systematic search of coccidioidomycosis clinical cases published from 1950 to 2021 was performed in the Scopus, PubMed, ScienceDirect, MEDLINE, and SciELO databases, using the keywords “coccidioidomycosis”, “treatment”, “clinical cases”, and “diagnosis”. The inclusion criteria were clinical cases that reported at least two parameters of the patient’s epidemiological data (age, gender, and geographical origin) as well as the diagnostic method and treatment. Conversely, the exclusion criteria were clinical cases with more than one parameter of the patient’s epidemiological data missing (age, gender, and geographical origin) and clinical cases that did not report the diagnostic method or treatment. Figure 1 illustrates the searching process.

The information obtained from the reviewed articles was organized in chronological order. For a better understanding, it was subdivided into tables with the patient’s epidemiological data (age, gender, and geographic origin of the patient). Patients with coccidioidomycosis were classified as having pulmonary or disseminated forms. Likewise, the diagnosis and treatment data were summarized in tables.

## 3. Results

The search yielded 81 articles on coccidioidomycosis clinical cases from 1950 to 2021. From this total, only 59 articles [17,40,41,42,43,44,45,46,47,48,49,50,51,52,53,54,55,56,57,58,59,60,61,62,63,64,65,66,67,68,69,70,71,72,73,74,75,76,77,78,79,80,81,82,83,84,85,86,87,88,89,90,91,92,93,94,95,96,97] that included 275 clinical cases and met the inclusion criteria were considered for this review. According to the information obtained, a higher incidence of coccidioidomycosis was shown in the male gender at 89.92% (247), while the female gender presented at 10.18% (28). Regarding age, patients with coccidioidomycosis were found in the age range from newborns to 80 years, with the most affected age group being 31–40 years, while the least impacted was 11–20 years (Table 2).

The clinical presentation of coccidioidomycosis corresponded primarily to disseminated coccidioidomycosis (67.64%), while the frequency of pulmonary coccidioidomycosis was 32.36%. It should be mentioned that within the disseminated cases of the disease, cutaneous and subcutaneous tissue involvement were the most predominant (61), followed by involvement of the CNS (42, generally in the form of meningitis), bone system (39), peritoneum (30), joints (20), lymphatic system (7), genitourinary system (6), eye (4), spleen (4), liver (3), heart (2), thyroid (2), muscle (1), adrenal glands (1), prostate (1), and placenta (1) (Table 3).

On the other hand, in those cases where the isolation of the fungus was possible, the species most frequently reported was *C. immitis*, followed by *Coccidioides* spp. Likewise, data from this review showed that the most typically used diagnostic methods were direct examination of biological samples with KOH and serological methods, mainly immunodiffusion, complement fixation, and, to a lesser extent, EIA (Table 4).

The results of this review showed that the most commonly used treatment for coccidioidomycosis were azoles (57.82%), followed by the combination of azoles and amphotericin B (17.1%), monotherapy with amphotericin B (12.73%), and the use of alternative medicine (4.36%) (Table 5).

At a deeper level, the most frequently used pharmacological regimen for treating pulmonary coccidioidomycosis was ketoconazole monotherapy (40), followed by fluconazole monotherapy (15), the combination of amphotericin B and ketoconazole (8), amphotericin B monotherapy (7), amphotericin B combined with fluconazole (6), itraconazole monotherapy (4), amphotericin B combined with itraconazole (2), voriconazole monotherapy (1), the combination of itraconazole and fluconazole (1), protoanemonin (1), and the use of non-steroidal anti-inflammatory drugs (1). Alternatively, the most widely used treatment for disseminated coccidioidomycosis was ketoconazole monotherapy (78), followed by the combination of amphotericin B and fluconazole (24), amphotericin B monotherapy (20), monotherapy with fluconazole (18), the combination of amphotericin B and ketoconazole (11), itraconazole monotherapy (7), amphotericin B combined with itraconazole (2), the combination of amphotericin B and azoles, a treatment not specified in the references (2), posaconazole monotherapy (1), fluconazole combined with itraconazole (1), and itraconazole combined with miconazole (1) (Table 2).

## 4. Discussion

This work reviews clinical cases of coccidioidomycosis published from 1950 to 2021 that provide epidemiological data, diagnostic methods, and treatment. The epidemiological data presented correspond mostly to cases reported in patients from the USA, showing how important this mycosis is in the country. In other countries, the publication of coccidioidomycosis clinical cases is not that relevant, and the reason behind this has not been clearly determined. In the USA, this mycosis is considered a public health problem. It is mandatory to notify in several states, mainly in endemic areas, which is not the case in other countries. For instance, in Mexico, since 1994, the disease has ceased to require mandatory notification, making epidemiological data scarce. In this review, only sixteen published works were found. That figure does not reflect the current situation of this mycosis, since other reports, which did not meet this review’s inclusion criteria, show an increased number of cases in the country. The same occurs in other countries of the American continent, such as Brazil, Venezuela, and Argentina, where this mycosis is considered significant [1]. In Mexico, specifically, we believe that this mycosis is very relevant since it shares the endemic geographic area of the fungus with the USA. Therefore, it should be expected to follow the same upward trend in the number of cases as the United States [3].

On the other hand, this review showed that the incidence of coccidioidomycosis was higher in the male gender than the female gender. Also, the most affected age group was 31–40 years, which coincides with data reported in Argentina, Venezuela, Brazil, and Mexico [4,5,98,99,100,101]. Likewise, the present work illustrated that the disseminated clinical form was the most predominant, mainly affecting cutaneous and subcutaneous tissue, the peritoneum, the CNS, and the bone system. However, it is worth mentioning that the primary pulmonary form, which in most cases resolves with a favorable outcome for the patient, is the most frequent clinical form of this mycosis and is not officially reported.

Furthermore, this work revealed that the most commonly used diagnostic methods for coccidioidomycosis were fungal identification through cultures and the complement fixation test. A previous study shows that serology has been maintained as the preferred method for an extended period [102], as well as obtaining cultures, even though coccidioidomycosis can be diagnosed through multiple tools. Notably, the complement fixation test, one of the most reported, is only performed in the USA. Unfortunately, this test has not been available for a long time in Central and South American countries, where cases of this mycosis have been described. In some countries, this test has been replaced by ELISA, using in-house antigens, which are also used to conduct gel immunodiffusion and capillary tube precipitation tests and are very useful for diagnosis, although few references document these assertions [103,104,105]. Likewise, other serological methods such as lateral-flow antibody assays [26], the detection of β-1,3-d-glucan [106], the use of recombinant antigens for antibody identification [107], the detection of coccidioidal metabolites in plasma and urine through specific liquid chromatography based on tandem mass spectrometry [108], as well as molecular methods, are not available in most countries or are not validated for routine use in the practice of mycological diagnosis, particularly molecular methods.

On the other hand, this work confirmed that the main therapeutic scheme for coccidioidomycosis is ketoconazole and amphotericin B, individually or in combination. However, ketoconazole appeared in the 1980s and was the first azole with demonstrable activity against *Coccidioides* spp. approved by the FDA for its treatment [39,109], so the cases reported in this work, during the period from 1980 to 1988, used ketoconazole as an antifungal agent choice; however, it is no longer used in the treatment of this mycosis due to the adverse effects it causes, including hepatotoxicity. Therefore, the cases published in subsequent years show that it was no longer used in the treatment of coccidioidomycosis, and only amphotericin B, fluconazole, itraconazole, and voriconazole were used (Table 2).

Nevertheless, some authors mention that no standard therapy effectively resolves the disease in all cases. Therefore, they suggest that the treatment of coccidioidomycosis should be highly individualized [38]. Also, it is essential to highlight that this treatment scheme applies mainly to cases diagnosed in the USA. In other countries, there is frequently no access to these antifungals due to their high cost, in addition to the fact that there is no reliable information on the number of coccidioidomycosis cases and how they are treated. Particularly in Mexico, this disease ceased to be notifiable in 1994, so there is no record of cases, and the few existing publications are restricted only to retrospective studies of this mycosis [110].

Currently, coccidioidomycosis poses a challenge with a significant impact on public health in countries where the disease is observed, mainly the United States of America, where more than 1,500,000 cases are registered per year (https://www.cdc.gov/fungal/diseases/coccidioidomycosis/statistics.html, accessed on 20 October 2023). Moreover, cases may be underreported in multiple countries on the American continent where this mycosis does not require a mandatory notification, aggravated by the fact that diagnostic tools are scarce.

On the other hand, although the literature highlights a significant advance in molecular methods as aids in diagnosing this mycosis, this study shows that they are not widely implemented as they are not available in all laboratories. More importantly, they are not validated methods. Also, therapeutic options are sometimes limited due to their high cost in several countries.

Despite efforts to develop diagnostic and therapeutic methods focused on preventing and combating coccidioidomycosis, even in countries like the USA, it continues to be a challenge and a public health problem. Meanwhile, the challenge is even greater in countries with fewer economic resources, as this mycosis is not even considered a public health problem. Therefore, sensitization has to be carried out among the health authorities in each country.

## 5. Conclusions

The analysis of clinical cases in Latin America shows that coccidioidomycosis has been increasing in recent years, and the geographical distribution of *Coccidioides* spp. is spreading. In other countries on the American continent, except for the United States, the threat of coccidioidomycosis is more insidious, as coccidioidomycosis is likely to be a much more significant threat than official or clinical records indicate. Therefore, continuous and extensive surveillance is necessary in the United States and the rest of the countries on the American continent to monitor trends and identify new potential areas of endemicity to inform public health authorities.

Likewise, this review reveals that the most frequently used diagnostic methods in daily medical practice are serological tests, especially the complement fixation test in the USA. In addition, culture remains the gold standard for diagnosing coccidioidomycosis, and the availability of these is probably due to socioeconomic conditions that do not allow the implementation of other more sensitive diagnostic techniques in other regions of the American continent.

In this work, the primary therapeutic regimen for coccidioidomycosis was ketoconazole and amphotericin B, individually or in combination. However, it should be considered that the use of ketoconazole was discontinued due to its hepatotoxic effects and replaced by other antifungals such as fluconazole, itraconazole, and voriconazole.

Based on the information obtained from this review, we consider that the development of new and faster diagnostic tools, as well as antifungal therapies directed at *Coccidioides* spp., is still necessary to advance the diagnosis and subsequent resolution of the disease, since knowledge about the epidemiology, diagnosis, and treatment of coccidioidomycosis can be used to guide future prevention and management strategies that minimize the morbidity and mortality caused by this mycosis.

## Figures and Tables

**Table 1:** Molecular methods used in the diagnosis of coccidioidomycosis.

Detection	Molecular Assay	Sample Target	Specific	Reference
*C. immitis*	Endpoint PCR	Fungal cultures	rRNA 28S	[28]
*C. immitis*	Endpoint PCR	Soil samples	ITS, rDNA	[29]
*C. imitis* and *C. posadasii*	Endpoint PCR	Clinical samples	Microsatellites GAC2 and 621	[30]
*C. immitis* and *C. posadasii*	Nested PCR	Tissue biopsies	DNA fragment of the Ag 2/*PRA* gene	[31]
*C. imitis* and *C. posadasii*	Endpoint PCR	Clinical isolates	From an rRNA sequence, random regions were selected, and their specificity was verified by PCR experiments for *C. imitis* and *C. posadasii*	[32]
*C. immitis* and *C. posadasii*	Endpoint PCR	Clinical isolates	From the sequences of the ITS regions of the rDNA (NS7-ITS2, ITS1-ITS4, ITS2-ITS5, ITS3-ITS4, ITS4-ITS5, ITS3-R635, and F63-R635), the oligonucleotides were selected, and only ITS3 and ITS4 differentiated the two species	[33]
*Coccidioides* spp.	Real-time PCR	Clinical samples	ITS2 region of *Coccidioides*	[34]
*Coccidioides* spp.	Endpoint PCR	Clinical samples	SCAR markers obtained from RAPD-PCR with random oligonucleotides	[35]
*C. immitis* and *C. posadasii*	Real-time PCR	Clinical samples	Modified from the conventional PCR of Umeyama et al. (2006); conserved regions of Ci45815 (GenBank No. AB597180.1) and Cp45810 (GenBank No. AB597183.1) flanking the excluded region for *C. posadasii*	[36]
*C. immitis* and *C. posadasii*	Next-generation metagenomic sequencing	Clinical samples	Sequencing of a large number of DNA fragments	[37]

**Table 2:** Clinical cases published from 1950 to 2021.

Reference	Reported Cases	Age	Gender	Country Where the Diagnosis Was Made	Geographic Origin of the Patient	Antecedent	Clinical Form	Diagnosis	Identified Species	Treatment
[40]	1	30	Female	United States of America	New York	Endemic area trip	Disseminated	CF + HP	*C. immitis*	Protoanemonin
[41]	1	27	Male	United States of America	California	Laborer hay field	Disseminated	Culture + HP + CF + PT	*C. immitis*	Amphotericin B
		43	Male	United States of America	Philippines	Endemic zone worker	Pulmonary	CF	*C. immitis*	Ethyl vanilla
		58	Male	United States of America	Valle de San Joaquín	Worker in endemic zone	Pulmonary	CF	*Coccidioides* spp.	Ethyl Vanilla/Isobutyl Vanillate
		28	Male	United States of America	California	ND	Pulmonary	Culture + CF	*C. immitis*	Ethyl Vanilla/Isobutyl Vanillate
[42]	8	54	Male	United States of America	Philippines	Worker in endemic zone	Pulmonary	ND	*Coccidioides* spp.	Ethyl vanilla
		27	Male	United States of America	Valle de San Joaquín	Worker in endemic zone	Disseminated	CF	*C. immitis*	Ethyl vanilla
		40	Male	United States of America	Valle de San Joaquín	ND	Disseminated	CF + PT	*Coccidioides* spp.	Ethyl vanilla
		34	Male	United States of America	Valle de San Joaquín	Worker in endemic zone	Disseminated	CF	*Coccidioides* spp.	Ethyl Vanilla/Isobutyl Vanillate
		22	Male	United States of America	Valle de San Joaquín	Worker in endemic zone	Pulmonary	CF	*Coccidioides* spp.	Ethyl vanilla
[43]	1	27	Female	Canada	Canada	Endemic area trip	Pulmonary	IDR + HP (G-G stain) + Culture	*C. immitis*	Nonsteroidal anti-inflammatory + Surgery
[44]	1	70	Male	United States of America	Canada	Worker in endemic zone	Pulmonary	PT + CF + Crop	*C. immitis*	Amphotericin B
[45]	1	50	Male	United States of America	Valle de San Joaquín	Cotton Field Worker and Grain Mill	Disseminated	Culture + DE + HP + CF	*C. immitis*	Amphotericin B + Miconazole
[46]	1	27	Male	United States of America	Arizona	Worker in endemic zone	Disseminated	IDR + CF + Culture	*C. immitis*	Amphotericin B
[47]	1	32	Male	United States of America	California	ND	Disseminated	CF	*Coccidioides* spp.	Amphotericin B + Miconazole
		79	Male	United States of America	ND	ND	Pulmonary	Culture + CF	*C. immitis*	Amphotericin B + Ketoconazole
		26	Male	United States of America	ND	ND	Pulmonary	Culture + CF	*C. immitis*	Ketoconazole
		66	Male	United States of America	ND	ND	Pulmonary	Culture + CF	*C. immitis*	Amphotericin B
		45	Male	United States of America	ND	ND	Pulmonary	IDR + CF + Culture	*C. immitis*	Amphotericin B + Ketoconazole
		52	Male	United States of America	ND	ND	Pulmonary	IDR + CF + Culture	*C. immitis*	Ketoconazole
		59	Male	United States of America	ND	ND	Pulmonary	Culture + CF	*C. immitis*	Amphotericin B + Ketoconazole
		31	Male	United States of America	ND	ND	Pulmonary	Culture + CF	*C. immitis*	Amphotericin B + Ketoconazole
[48]		32	Male	United States of America	ND	ND	Pulmonary	Culture + CF	*C. immitis*	Amphotericin B + Ketoconazole
	18	47	Female	United States of America	California	ND	Pulmonary	Culture + CF	*C. immitis*	Ketoconazole
		66	Male	United States of America	ND	ND	Pulmonary	Culture + CF	*C. immitis*	Amphotericin B + Ketoconazole
		34	Male	United States of America	ND	ND	Disseminated	Culture + CF	*C. immitis*	Amphotericin B + Ketoconazole
		31	Male	United States of America	ND	ND	Disseminated	Culture + CF	*C. immitis*	Amphotericin B + Ketoconazole
		35	Male	United States of America	ND	ND	Disseminated	Culture + CF	*C. immitis*	Ketoconazole
		28	Male	United States of America	ND	ND	Disseminated	Culture + CF	*C. immitis*	Amphotericin B + Ketoconazole
		39	Male	United States of America	ND	ND	Disseminated	Culture + CF	*C. immitis*	Amphotericin B + Ketoconazole
		73	Male	United States of America	ND	ND	Disseminated	IDR + CF + Crop	*C. immitis*	Amphotericin B + Ketoconazole
		35	Male	United States of America	ND	ND	Disseminated	CF	*C. immitis*	Amphotericin B + Ketoconazole
		57	Male	United States of America	ND	ND	Disseminated	Culture + CF	*C. immitis*	Amphotericin B + Ketoconazole
[49]		40–44	Male	United States of America	ND	ND	Disseminated (77)	HP + Culture	*C. immitis*	Ketoconazole
	112			United States of America		ND	Pulmonary (35)			
		51	Male	United States of America	ND	ND	Disseminated	Culture + CF	*Coccidioides* spp.	Amphotericin B + Fluconazole
		44	Male	United States of America	ND	ND	Disseminated	Culture + CF	*Coccidioides* spp.	Amphotericin B + Fluconazole
[50]		33	Male	United States of America	ND	ND	Disseminated	Culture + CF	*Coccidioides* spp.	Amphotericin B + Fluconazole
	8	71	Male	United States of America	ND	ND	Disseminated	Culture + CF	*Coccidioides* spp.	Amphotericin B + Fluconazole
		39	Female	United States of America	ND	ND	Pulmonary	Culture + CF	*Coccidioides* spp.	Amphotericin B + Flucytosine
		32	Male	United States of America	ND	ND	Pulmonary	Culture + CF	*Coccidioides* spp.	Amphotericin B + Fluconazole
		55	Male	United States of America	ND	ND	Disseminated	Culture + CF	*Coccidioides* spp.	Amphotericin B + Fluconazole
		46	Male	United States of America	ND	ND	Disseminated	Culture + CF	*Coccidioides* spp.	Amphotericin B + Fluconazole
[51]	1	33	Male	Colombia	Colombia	Endemic area trip	Disseminated	DE (KOH) + Culture + ID + CF	*C. immitis*	Amphotericin B
[52]	1	42	Male	United States of America	United States of America	Endemic area trip	Disseminated	HP (PAS) + Culture+ CF	*C.immitis*	Amphotericin B
[53]	2	17	Male	Mexico	Coahuila	ND	Pulmonary	IDR + HP	*C.immitis*	Ketoconazole
		25	Male	Mexico	Chihuahua	ND	Pulmonary	DE (KOH) + Culture	*C.immitis*	Ketoconazole
	3	42	Male	United States of America	California	ND	Disseminated	CF	*C. immitis*	Fluconazole
[54]		18	Female	United States of America	California	ND	Disseminated	HP + CF	*C. immitis*	Fluconazole
		33	Male	United States of America	California	ND	Disseminated	CF	*C. immitis*	Amphotericin B + Fluconazole
[55]	1	30	Male	United States of America	California	ND	Disseminated	CF + HP (G-G staining)	*C. immitis*	Amphotericin B
[56]	1	27	Female	United States of America	New Mexico	Endemic area trip	Disseminated	HP + HR	*C. immitis*	Amphotericin B + Fluconazole
[57]	1	76	Female	United States of America	Pakistan	Endemic area trip	Disseminated	HP (PAS + G-G stain)	*C. immitis*	ND
[58]	2	24	Male	United States of America	California	Military	Pulmonary	Culture + (Gram stain)	*C. immitis*	ND
		64	Male	United States of America	Arizona	Worker in endemic zone	Pulmonary	Culture + CF + ID	*C. immitis*	Amphotericin B
[59]	1	36	Male	United States of America	Philippines	ND	Disseminated	Culture + Smear	*C. immitis*	Amphotericin B + Fluconazole
		45	Male	United States of America	California	Migrant	Disseminated	Culture + CF	*Coccidioides* spp.	Amphotericin B + Fluconazole
[60]		21	Male	United States of America	California	ND	Pulmonary	DE (KOH) + CF+ Culture + PTC + HP	*C. immitis*	Amphotericin B + Fluconazole
		36	Male	United States of America	Calfornia	ND	Disseminated	------------	*C. immitis*	Fluconazole
	6	42	Male	United States of America	California	ND	Disseminated	HP + CF	*C. immitis*	Amphotericin B + Fluconazole
		63	Male	United States of America	New York	Worker in endemic zone	Disseminated	HP + CF	*C. immitis*	Amphotericin B + Fluconazole
		50	Male	United States of America	Valle de San Joaquín	Endemic area trip	Disseminated	HP + Culture + CF + ID	*C. immitis*	Amphotericin B + Fluconazole
[61]	1	52	Male	ND	ND	ND	Pulmonary	CF + LA	*C. immitis*	Amphotericin B + Ketoconazole
[62]	1	52	Male	Colombia	Colombia	Worker in endemic zone	Pulmonary	DE + Culture + HP + CF + ID	*Coccidioides* spp.	Itraconazole
[63]	2	32	Male	United States of America	California	Convict	Disseminated	HP	*Coccidioides* spp.	Amphotericin B
		31	Female	-------------	ND	ND	Disseminated	HP + Culture	*C. immitis*	Amphotericin B + Fluconazole
[64]	1	38	Male	United States of America	California	ND	Disseminated	DE (KOH) + PCR + Culture	*C. immitis*	Amphotericin B + Fluconazole
[17]	1	80	Male	United States of America	California	Endemic area trip	Pulmonary	HP (H-E and G-G stain) + immunofluorescence	*C. immitis*	Amphotericin B + Fluconazole
[65]	2	23 days	Male	United States of America	Philippines	Contact with dust	Pulmonary	CF	*C. immitis*	Amphotericin B + Fluconazole
		25 s 2 d	Female	United States of America	California	Maternal history	Pulmonary	PCR	*C. immitis*	Amphotericin B
		34	Male	Mexico	Coahuila	ND	Pulmonary	Culture + DE (KOH)	*C.immitis*	Fluconazole
[66]		42	Male	Mexico	Coahuila	ND	Pulmonary	Culture + DE (KOH)	*C.immitis*	Amphotericin B + Fluconazole
	6	23	Male	Mexico	Coahuila	ND	Pulmonary	Culture + DE (KOH)	*C.immitis*	Amphotericin B
		62	Male	Mexico	Coahuila	ND	Pulmonary	DE	*C.immitis*	Fluconazole
		28	Male	Mexico	Coahuila	ND	Pulmonary	Culture + DE (KOH)	*C.immitis*	Fluconazole
		22	Male	Mexico	Coahuila	ND	Pulmonary	HP	*C.immitis*	Amphotericin B + Fluconazole
[67]	1	45	Female	United States of America	Texas	Migrant	Disseminated	DE + Culture + CF + IDR	*C. immitis*	Fluconazole
[68]	1	RN	Female	ND	H	Maternal history	Pulmonary	CF + Culture + PCR	*C. immitis*	Amphotericin B + Fluconazole
[69]	1	11	Male	ND	ND	ND	Pulmonary	HP + Culture + CF	*C. immitis*	Amphotericin B + Fluconazole
		41	Female	ND	ND	Endemic area trip	Disseminated	HP + Culture + CF	*C. immitis*	Amphotericin B + Ketoconazole
[70]		48	Female	ND	Pakistan	Endemic area trip	Pulmonary	HP + Culture + CF + ID	*C. immitis*	Amphotericin B + Itraconazole
	6	38	Male	United States of America	Arizona	ND	Pulmonary	HP + CF + ID	*C. immitis*	Amphotericin B + Itraconazole
		41	Male	United States of America	Arizona	ND	Pulmonary	Culture + HP	*C. immitis*	Amphotericin B
		27	Male	United States of America	Arizona	ND	Pulmonary	Culture + CF + ID + HP	*C. immitis*	Amphotericin B
		54	Female	United States of America	Philippines	Lives in endemic area	Pulmonary	HP (G-G stain) + CF	*C. immitis*	Amphotericin B + Fluconazole
[71]	2	74	Male	ND	ND	ND	Disseminated	HP	*C. immitis*	Amphotericin B + Ketoconazole
		33	Male	ND	ND	ND	Disseminated	HP	*C. immitis*	Fluconazole
		71	Female	Canada	Canada	Endemic area trip	Disseminated	Culture	*C. immitis*	Amphotericin B + Fluconazole
		35	Male	Canada	Japan	ND	Disseminated	Culture + HP	*Coccidioides* spp.	ND
		48	Male	Canada	ND	ND	Disseminated	CF + HP + PTC	*Coccidioides* spp.	Amphotericin B
		30	Male	Canada	ND	ND	Disseminated	HP + CF + IDR	*Coccidioides* spp.	ND
		5	Male	Canada	ND	ND	Disseminated	HP + HR	*Coccidioides* spp.	Amphotericin B
		37	Male	Canada	Japan	ND	Disseminated	CF	*Coccidioides* spp.	Amphotericin B
		16	Male	Canada	ND	ND	Disseminated	HP + CF	*Coccidioides* spp.	ND
		48	Female	Canada	ND	ND	Disseminated	CF + IDR	*Coccidioides* spp.	ND
		32	Male	Canada	H	ND	Disseminated	------------	*Coccidioides* spp.	ND
		24	Male	Canada	ND	ND	Disseminated	CF + PTC	*Coccidioides* spp.	Amphotericin B
		35	Male	Canada	ND	ND	Disseminated	Culture + CF	*Coccidioides* spp.	Amphotericin B
[72]		30	Female	Canada	ND	ND	Disseminated	Culture + CF + HP	*Coccidioides* spp.	ND
	27	34	Male	Canada	ND	ND	Disseminated	CF + HP	*Coccidioides* spp.	Amphotericin B
		30	Male	Canada	ND	ND	Disseminated	CF + HP	*Coccidioides* spp.	Amphotericin B
		38	Female	Canada	ND	ND	Disseminated	Culture + CF	*Coccidioides* spp.	ND
		36	Male	Canada	ND	ND	Disseminated	Culture + CF + HP	*Coccidioides* spp.	Amphotericin B
		20	Female	Canada	ND	ND	Disseminated	CF + HP	*Coccidioides* spp.	ND
		16	Male	Canada	ND	ND	Disseminated	CF + HR	*Coccidioides* spp.	ND
		7	Male	Canada	ND	ND	Disseminated	Culture	*Coccidioides* spp.	ND
		25	Female	Canada	ND	ND	Disseminated	CF + Culture	*Coccidioides* spp.	ND
		77	Male	Canada	Canada	ND	Disseminated	HP + CF	*Coccidioides* spp.	ND
		28	Male	Canada	Japan	ND	Disseminated	HP + CF + IDR	*Coccidioides* spp.	Amphotericin B + Ketoconazole
		21	Male	Canada	Canada	ND	Disseminated	Culture + CF + HP + PTC	*Coccidioides* spp.	Amphotericin B + Ketoconazole
		57	Male	Canada	Canada	ND	Disseminated	Culture + IDR	*C. immitis*	Amphotericin B
		42	Male	Canada	Canada	ND	Disseminated	Culture + CF + HP	*Coccidioides* spp.	Amphotericin B
		30	Male	Canada	Canada	ND	Disseminated	HP + CF	*Coccidioides* spp.	Amphotericin B
		71	Female	Canada	Canada	ND	Disseminated	Culture + HP	*Coccidioides* spp.	Amphotericin B + Fluconazole
[73]		46	Female	ND	ND	Endemic area trip	Pulmonary	Culture + CF + HP (H-E and G-G staining)	*C. immitis*	Amphotericin B + Itraconazole
		56	Male	United States of America	ND	ND	Disseminated	CF + Smear (Gram) + Culture	*C. immitis*	Amphotericin B + Itraconazole
	5	48	Male	ND	ND	ND	Disseminated	CF + Culture + Smear (Gram)	*C. immitis*	Amphotericin B + Itraconazole
		45	Male	ND	ND	ND	Disseminated	CF + DE + Crop	*C. immitis*	Amphotericin B + Itraconazole
		--------------	Male	United States of America	California	Endemic area trip	Disseminated	ID + CF	*C. immitis*	Amphotericin B + Itraconazole
[74]	1	41	Male	Paraguay	Paraguay	Travel to non-endemic area	Pulmonary	DE	*C. immitis*	Amphotericin B + Fluconazole
[75]	2	3	Male	Mexico	Sonora	ND	Disseminated	Culture + CF + ED	*C. immitis*	Fluconazole
		3	Male	Mexico	Mexico	ND	Disseminated	CF + ID	*Coccidioides* spp.	Fluconazole
[76]	2	53	Male	United States of America	California	ND	Disseminated	HP + CF	*C. immitis*	Fluconazole
		78	Male	United States of America	Texas	Endemic area trip	Disseminated	HP (H-E stain) + CF	*C. immitis*	Itraconazole
[77]	1	44	Male	ND	ND	ND	Disseminated	HP + CF + EIA	*Coccidioides* spp.	ND
[78]	1	38	Male	United States of America	Poland	Endemic area trip	Pulmonary	CF	*C. immitis*	ND
[79]	1	58	Male	United States of America	AS	Endemic area trip	Pulmonary	HP (H-E and G-G stain)	*C. immitis*	Fluconazole
		47	Male	United States of America	Texas	ND	Pulmonary	CF + Culture	*Coccidioides* spp.	Fluconazole
		36	Male	United States of America	Texas	ND	Pulmonary	CF + Culture	*Coccidioides* spp.	Itraconazole + Fluconazole
		37	Male	United States of America	Texas	ND	Pulmonary	CF + Culture	*Coccidioides* spp.	ND
[80]		36	Male	United States of America	Texas	ND	Pulmonary	CF + Culture	*Coccidioides* spp.	Fluconazole
	9	39	Male	United States of America	Texas	ND	Pulmonary	CF + Culture	*Coccidioides* spp.	Itraconazole
		55	Male	United States of America	Mexico	ND	Pulmonary	CF + Culture	*Coccidioides* spp.	Fluconazole
		24	Male	Mexico	Mexico	ND	Pulmonary	CF + Culture	*Coccidioides* spp.	Fluconazole
		73	Male	Mexico	Mexico	ND	Pulmonary	CF + Culture	*Coccidioides* spp.	ND
		33	Female	Mexico	Mexico	ND	Pulmonary	CF + Culture	*Coccidioides* spp.	Itraconazole
[81]	2	5	Female	Mexico	Tijuana	ND	Pulmonary	HP + Culture + CF	*C. immitis*	Fluconazole
		9	Male	Mexico	Sinaloa	ND	Disseminated	HP (G-G stain) + Culture	*C. immitis*	Itraconazole
[82]	1	31	Male	United States of America	India	Endemic area trip	Disseminated	DE (PAS + LF) + Culture + PCR + CF	*C. posadasii*	Amphotericin B + Itraconazole
[83]	1	52	Male	Mexico	San LuisPotosí	Worker in endemic zone	Disseminated	HP (PAS + G-G)	*Coccidioides* spp.	Fluconazole
[84]	1	54	Male	United States of America	Texas	ND	Disseminated	HP	*Coccidioides* spp.	Fluconazole
[85]	1	63	Male	United States of America	Arizona	ND	Disseminated	CF	*Coccidioides* spp.	Fluconazole
		50	Male	Mexico	Guanajuato	Worker in endemic zone	Disseminated	DE (KOH) + Culture	*Coccidioides* spp.	Itraconazole
[86]		28	Male	Mexico	Mexico	Worker in endemic zone	Disseminated	Culture + IDR	*C. posadasii*	Itraconazole
	6	59	Male	Mexico	Michoacán	Worker in endemic zone	Disseminated	DE (KOH) + Culture + IDR	*C. posadasii*	Amphotericin B + Itraconazole
		34	Male	Mexico	Zacatecas	Spider bite	Disseminated	HP + Culture	*Coccidioides* spp.	Itraconazole
		32	Male	Mexico	Michoacán	Worker in endemic zone	Disseminated	DE (KOH) + Culture	*C. posadasii*	Amphotericin B
		48	Male	Mexico	Puebla	Worker in endemic zone	Disseminated	DE (KOH) + Culture	*Coccidioides* spp.	Itraconazole
[87]	1	28	Female	ND	--------------	ND	Pulmonary	HP	*C. immitis*	Amphotericin B
[88]	1	33	Male	United States of America	Guatemala	Worker in endemic zone	Disseminated	HP (H-E and G-G stain) + PCR	*C. immitis*	Itraconazole
[89]		32	Male	Brazil	Brazil	Armadillo hunter	Pulmonary	DE (KOH)	*Coccidioides* spp.	Fluconazole
	3	40	Male	Brazil	Brazil	Armadillo hunter	Pulmonary	DE (KOH)	*Coccidioides* spp.	Fluconazole
		71	Male	Brazil	Brazil	Armadillo hunter	Disseminated	DE (KOH)	*Coccidioides* spp.	Fluconazole
[90]	1	8	Male	Mexico	Chihuahua	Worker in endemic zone	Pulmonary	HP	*Coccidioides* spp.	Voriconazole
[91]	1	62	Male	United States of America	Arizona	ND	Disseminated	EIA	*Coccidioides* spp.	Fluconazole
[92]	1	27	Male	United States of America	Philippines	ND	Disseminated	HP + CF + ID	*Coccidioides* spp.	Amphotericin B
[93]	1	38	Male	ND	ND	ND	Disseminated	HP (H-E stain) + Culture + CF + EIA	*Coccidioides* spp.	Posaconazole
		37	Female	ND	H	ND	Pulmonary	CF	*C. immitis*	Fluconazole
[94]		63	Male	ND	H	ND	Pulmonary	CF	*C. immitis*	Fluconazole
	5	42	Female	ND	H	ND	Pulmonary	CF	*C. immitis*	Fluconazole
		45	Male	ND	H	ND	Pulmonary	CF	*C. immitis*	Fluconazole
		35	Male	ND	H	ND	Pulmonary	CF + ID	*C. immitis*	Fluconazole
[95]	1	4	Male	United States of America	California	Residence in endemic area	Pulmonary	HP (H-E stain) + PCR + CF	*Coccidioides* spp.	Amphotericin B + Fluconazole
[96]	1	24	Female	United States of America	New Mexico	ND	Disseminated	CF+HP (H-E stain, G-G)	*Coccidioides* spp.	Fluconazole
[97]	1	44	Male	United States of America	H	Travel to endemic area; inmate in a prison in Texas	Pulmonary	ID + CF + Culture + HP (H-E staining)	*C. immitis*	Amphotericin B + Fluconazole

CF: complement fixation; HP: histopathology; PT: tube precipitation test; IDR: intradermal reaction; G-G: Gomori–Grocott methanamine silver; DE: direct examination; PAS: periodic acid–Schiff; H-E: hematoxylin–eosin; PCR: polymerase chain reaction; ID: immunodiffusion; LF: lactophenol blue; EIA: immunoenzymatic assay; H: Hispanic (country not defined); AS: Asian (country not defined); ND: undefined.

**Table 3:** Frequency of affected anatomical sites in patients with disseminated coccidioidomycosis.

Anatomical Site Affected	Number of Cases	Frequency (%)
Skin and subcutaneous tissue	61	28.24
Central Nervous System	42	18.98
Bone	32	14.81
Peritoneum	30	13.88
Joints	20	9.25
Lymphatic system	7	3.24
Genitourinary system	6	2.77
Eye	4	1.85
Spleen	4	1.85
Liver	3	1.38
Heart	2	0.92
Muscle	1	0.46
Adrenal glands	1	0.46
Thyroid glands	1	0.46
Prostate	1	0.46
Placenta	1	0.46

**Table 4:** Laboratory diagnostic tests.

Microbiological Methods	Number of Cases
Culture	203
Histopathology (H-E; G-G; PAS)	175
Direct examination	23
Smear	4
Serological Methods	
CF (IgG)	117
Intradermal reaction	13
ID	11
PT (IgM)	6
EIA (IgM-IgG)	3
Latex agglutination	1
Immunofluorescence	1
Molecular Method	
PCR	6

H-H: hematoxylin–eosin; G-G: Gomori–Grocott; PAS: periodic acid–Schiff; CF: complement fixation; ID: immunodiffusion; PT: tube precipitation test; EIA: immunoenzymatic assay; PCR: polymerase chain reaction.

**Table 5:** Treatment scheme used in cases of coccidioidomycosis.

Drug	Number of Cases	Frequency (%)
Azoles	158	57.45
Amphotericin B + azoles	47	17.1
Amphotericin B	36	13.09
Other	12	4.36
Not specified	22	8.0
Total	275	100

## Data Availability

Not applicable.
